# Pamidronate decreases bilirubin-impaired cell death and improves dentinogenic dysfunction of stem cells from human deciduous teeth

**DOI:** 10.1186/s13287-018-1042-7

**Published:** 2018-11-08

**Authors:** Haruyoshi Yamaza, Soichiro Sonoda, Kazuaki Nonaka, Toshio Kukita, Takayoshi Yamaza

**Affiliations:** 1 Department of Pediatric Dentistry, Division of Oral Health, Growth and Development, Kyushu University Graduate School of Dental Science, 3-1-1 Maidashi, Higashi-ku, Fukuoka, 812-8582 Japan; 2 Department of Molecular Cell Biology and Oral Anatomy, Division of Oral Biological Sciences, Kyushu University Graduate School of Dental Science, 3-1-1 Maidashi, Higashi-ku, Fukuoka, 812-8582 Japan

**Keywords:** Stem cells from human exfoliated deciduous teeth (SHED), Bilirubin, Pamidronate, Cell death

## Abstract

**Background:**

Hyperbilirubinemia that occurs in pediatric liver diseases such as biliary atresia can result in the development of not only jaundice in the brain, eyes, and skin, but also tooth abnormalities including green pigmentation and dentin hypoplasia in the developing teeth. However, hyperbilirubinemia-induced tooth impairments remain after liver transplantation. No effective dental management to prevent hyperbilirubinemia-induced tooth impairments has been established.

**Methods:**

In this study, we focused on pamidronate, which is used to treat pediatric osteopenia, and investigated its effects on hyperbilirubinemia-induced tooth impairments. We cultured stem cells from human exfoliated deciduous teeth (SHED) under high and low concentrations of unconjugated bilirubin in the presence or absence of pamidronate. We then analyzed the effects of pamidronate on the cell death, associated signal pathways, and dentinogenic function in SHED.

**Results:**

We demonstrated that a high concentration of unconjugated bilirubin induced cell death in SHED via the mitochondrial pathway, and this was associated with the suppression of AKT and extracellular signal-related kinase 1 and 2 (ERK1/2) signal pathways and activation of the nuclear factor kappa B (NF-κB) signal pathway. The high concentration of unconjugated bilirubin impaired the in vitro and in vivo dentinogenic capacity of SHED, but not the low concentration. We then demonstrated that pamidronate decreased the bilirubin-induced cell death in SHED via the altered AKT, ERK1/2, and NF-κB signal pathways and recovered the bilirubin-impaired dentinogenic function of SHED.

**Conclusions:**

Our findings suggest that pamidronate may prevent tooth abnormalities in pediatric patients with hyperbilirubinemia.

**Electronic supplementary material:**

The online version of this article (10.1186/s13287-018-1042-7) contains supplementary material, which is available to authorized users.

## Background

Stem cells from human exfoliated deciduous teeth (SHED) have been identified as a sub-population of highly proliferative mesenchymal stem cells (MSCs) with self-renewal capacity and multi-differentiation potential [1, 2]. Given the critical ability of SHED to form dentin [3, 4], SHED are considered as the ideal dentinogenic stem cells to investigate the cellular and molecular mechanisms of dentinogenesis in both physiological and pathological processes of developing teeth.

Pediatric hyperbilirubinemia, which is caused by congenital liver disorders, such as congenital biliary atresia (BA) and Wilson disease, results in the development of jaundice in the brain, eyes, and skin [5, 6]. The excess blood bilirubin impairs osteogenic function and cell viability of bone-forming cells [7, 8], resulting in secondary osteoporosis of the growing skeletal system [9, 10]. Meanwhile, pediatric hyperbilirubinemia injures developing deciduous and permanent dentition associated with green pigmentation and dentin hypoplasia [11, 12], which raises the risk of dental caries. However, the underlying mechanisms of dentin hypoplasia by hyperbilirubinemia remain unclear. A recent in vitro study demonstrated that bilirubin induced cell death and dentinogenic dysfunction in SHED via alterations in AKT extracellular-regulated kinases 1/2 (ERK1/2) and nuclear factor kappa B (NF-κB) signal pathways [13].

Once pediatric patients with refractory liver diseases receive a liver transplantation, osteoporosis is improved [14, 15]. However, although impaired oral findings associated with hyperbilirubinemia and liver transplantation were reported [16, 17], the underlining mechanisms and the appropriated treatments for their associated oral impairments including dentin hypoplasia have not been investigated fully, suggesting that the tooth abnormalities may undermine the subsequent healthy development and quality of life in pediatric patients with hyperbilirubinemia. However, no effective dental care to prevent hyperbilirubinemia-induced tooth injuries has been established. Bisphosphonates are used as a safe and useful pharmacological approach for pediatric patients with osteogenesis imperfecta and secondary osteoporosis in BA and Wilson disease [18, 19]. Pamidronate provides the clinical benefits of preventing bone loss and bone fracture in pediatric patients awaiting liver transplantation [20]. However, the pharmacological efficacy of pamidronate in tooth failure in pediatric hyperbilirubinemia has not been investigated.

SHED are maintained by multiple pathways including the AKT, ERK1/2, and NF-κB signal pathways [2]. The dentinogenic capacity of SHED is regulated by multiple pathways including the AKT, ERK1/2, and NF-κB signal pathways [21–23]. In this study, it aimed to develop a novel pharmacological treatment or prevention for a tooth failure in patients with hyperbilirubinemia. We then examined the cell death, dentinogenic capacity, and the associated intracellular signal pathways including AKT, ERK1/2, and NF-κB in SHED under excess bilirubin exposure in vitro. We further investigated the expression of mitochondrial-cell death pathway-related molecules including BCL2, cytochrome c, and caspase 3. To estimate the ability to prevent tooth failure using a pharmacological approach, we also investigated the treatment efficacy of pamidronate on the bilirubin-induced dysfunction to cell death and dentinogenesis and the associated molecules in SHED.

## Methods

### Ethical statement and human subjects

Human deciduous teeth were collected as discarded biological/clinical samples from healthy pediatric donors (5–7 years old) in the Department of Pediatric Dentistry of Kyushu University Hospital. Procedures using human samples were approved by the Kyushu University Institutional Review Board for Human Genome/Gene Research (Protocol Number: 678-00). We obtained written informed consent from the parents on behalf of the child donors. All animal experiments were approved by the Institutional Animal Care and Use Committee of Kyushu University (Protocol Number: A21-044-1).

### Isolation, culture, and characterization of SHED

SHED were isolated and cultured as described in the Additional file 1 according to previous reports [1, 2]. Passage 3 SHED were used for further experiments.

### Preparation and treatment of bilirubin and pamidromate solutions

Unconjugated bilirubin (Merk, Darmstadt, Germany) was diluted as according to the previous report [13]. Pamidronate (Tokyo Chemical Industry, Tokyo, Japan) was diluted in distilled phosphate buffered saline (PBS). In this study, we examined three group treated with 0 μM bilirubin, 50 μM bilirubin, and 50 μM bilirubin plus 10 μM pamidronate. Control cultures were treated with the same amount of NaOH and albumin without bilirubin and/or PBS.

### Cell viability assay

SHED (10 × 10^4^/well) were seeded on 96-well multiplates and were incubated 24 h. Then, the cells were cultured with 0 μM bilirubin, 50 μM bilirubin (Merck), and 50 μM bilirubin (Merck) plus 10 μM pamidronate (Tokyo Chemical Industry) in the absence of serum for 3 days. Viability of the cells was assayed using a Cell Counting Kit-8 (Dojindo, Kumamoto, Japan) according to the manufacturer’s instructions and was measured at OD 450 nm on a plate-reader Multiscan GO (Thermo Scientific, Walthan, MA).

### Cell death assay

SHED were seeded at 10 × 10^3^ and 100 × 10^3^ cells per dish on 35-mm and 60-mm culture dishes, respectively, and were incubated for 24 h. Then, the cells were cultured with 0 μM bilirubin, 50 μM bilirubin (Merck), and 50 μM bilirubin (Merck) plus 10 μM pamidronate (Tokyo Chemical Industry) in the absence of serum for 3 days. For the terminal deoxynucleotidyl transferase (TdT)-mediated dUTP nick end labeling (TUNEL) assay, the cells were treated with the ApoTag Peroxidase In Situ Apoptosis Detection kit (Merck) according to the manufacturer’s instructions. The results are represented as a percentage of the numbers of the TUNEL-positive nuclei to the total ones with nucleated cells as described previously [13]. Moreover, the cells were stained with R-phycoerythrin-conjugated Annexin-V (eBioscience, San Diego, CA) and 7AAD (eBioscience) according to the manufacturer’s instructions and were analyzed with a FACS Verse flow cytometer (BD Bioscience, Franklin Lake, NJ).

### Real-time reverse transcription polymerase chain reaction (RT-PCR) assay

Real-time RT-PCR assays were performed as described in Additional file 1.

### Western blot analysis

Western blot analysis was performed as described in Additional file 1.

### Nuclear translocation assay of NF-kB

SHED were seeded at 1 × 10^3^ on 8-well chamber slides and were cultured for 24 h. The cells were incubated for 8 h under a serum-depleted condition and were subsequently stimulated with 0 μM bilirubin, 50 μM bilirubin (Merck), and 50 μM bilirubin (Merck) plus 10 μM pamidronate (Tokyo Chemical Industry) in the absence of serum for 3 days. As a control, SHED were treated with tumor necrosis factor alpha (TNFα; 10 nM, Peprotech, Rocky Hill, NJ). The cultures were fixed with 4% paraformaldehyde in PBS and analyzed by immunofluorescent microscopy.

### In vitro dentinogenic assay

SHED (10 × 10^4^ per 60 mm culture dish) were cultured under dentinogenic conditions with 0 μM bilirubin, 50 μM bilirubin (Merck), and 50 μM bilirubin plus 10 μM pamidronate (Tokyo Chemical Industry) according to previous studies [13]. Two and 4 weeks after the dentinogenic induction, the cultures were harvested for the examination of gene and protein expression and calcium deposition, respectively.

### Calcium deposition assay

Calcium deposition was assayed by alizarin red staining as described previously [13].

### In vivo tissue formation assay

SHED were pretreated with 0 μM bilirubin, 50 μM bilirubin (Merck), and 50 μM bilirubin plus 10 μM pamidronate (Tokyo Chemical Industry) for 7 days. The cells (4 × 10^6^) were mixed with hydroxyapatite tricalcium phosphate (40 mg, Zimmer Inc., Warsaw, IN) and were subcutaneously transplanted into 8- to 10-week-old female Balb/c nude/nude (Japan CLEA, Shizuoka, Japan) as described previously [2]. The implants were harvested 4 weeks after the transplantation. Frozen sections were treated for hematoxylin and eosin staining or immunofluorescent microscopy. The mineralized tissue area is represented as a percentage of the total area as described previously [2].

### Immunofluorescent microscopy

Immunofluorescent microscopy was performed as described in Additional file 1.

### Statistical analysis

All data are expressed as the mean ± standard error of the mean (SEM) or the mean ± standard deviation (SD) of at least triplicate measurements. Comparisons between two groups were analyzed using independent two-tailed Student’s *t* tests. Multi-group comparisons were analyzed using one-way repeated measures analysis of variance followed by Tukey’s post hoc test. *P* values less than 0.05 were considered as statistically significant. Statistical analysis was performed using a PRISM 6 software (GraphPad, Software, La Jolla, CA).

## Results

### Bilirubin induces apoptosis in SHED

**Fig. 1:**
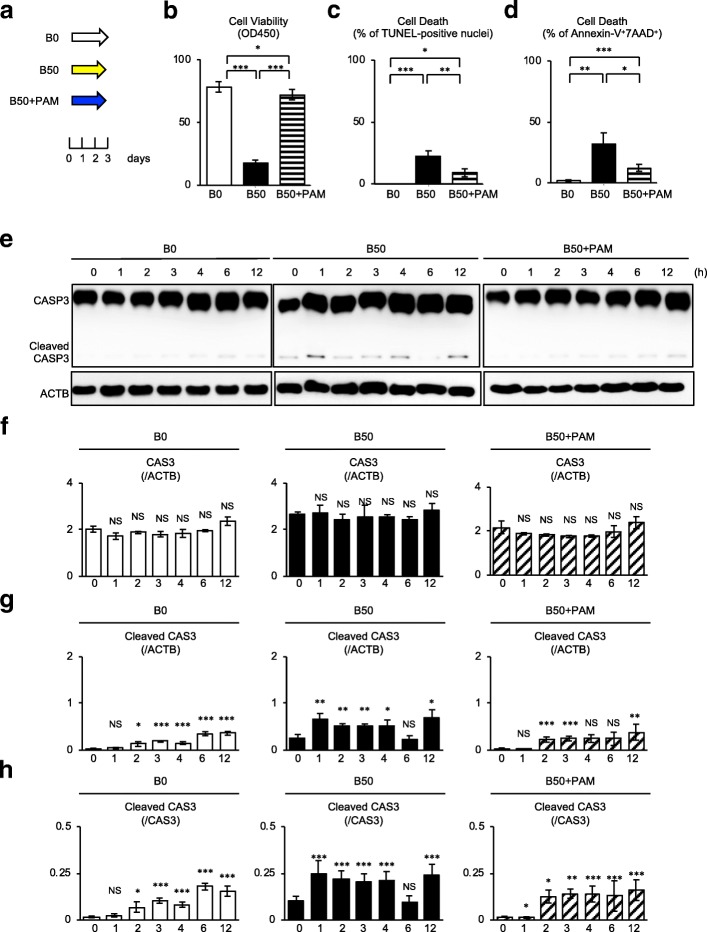
Pamidronate suppresses cell death and reverses the altered expression of caspase 3 and its cleaved in bilirubin-impaired SHED. **a** A scheme of pamidronate treatment of bilirubin-impaired SHED. SHED were cultured with 0 μM bilirubin (B0), 50 μM bilirubin (B50), and 50 μM bilirubin plus 10 μM pamidronate (B50+PAM) under serum-depleted condition for 3 days. **b** Cell viability analysis was performed after 3 days of the culture. **c**, **d** Cell death assays. Terminal deoxynucleotidyl transferase (TdT)-mediated dUTP nick end labeling (TUNEL) staining was performed after 3 days of the culture (**c**). Flow cytometric assay with Annexin-V (AV) and 7AAD staining was performed after 3 days of the culture (**d**). **e**–**h** Sequential expression of caspase 3 (CAS3) and the cleaved caspase 3 (Cleaved CAS3) was analyzed at the indicated time by western blot analysis. Representative images of western blotting were shown (**e**). Results were shown as the representative expression of CASP3 to beta-actin (ACTB) (**f**), Cleaved CAS3 to ACTB (**g**), and Cleaved CAS3 to CAS3 (**h**) at each time point in each group. **b**–**d**, **f**–**h** *n* = 5 for all groups. Statistical analysis was performed as described in the “Methods” section. Graph bars showed the means ± SEM. **b**–**d** *P < 0.05, **P < 0.01, and ***P < 0.005. **f**–**h** *P < 0.05, **P < 0.01, and ***P < 0.005 (vs. 0 h in each group). NS, no significance

A recent study demonstrated that unconjugated bilirubin (50 μM) induces cell death in SHED, but the bilirubin concentration was not specified [13]. In this study, we examined the concentration-dependent effects on cell death in SHED. SHED were cultured in different concentrations of unconjugated bilirubin (0, 10, and 50 μM) under serum-depleted conditions (Additional file 2: Figure S1a). The viability of SHED stimulated with 50 μM unconjugated bilirubin, B50-SHED, significantly decreased in a time-dependent manner, especially on days 2 and 3, but this effect was not observed in SHED stimulated with 0 μM and 10 μM unconjugated bilirubin, B0-SHED and B10-SHED, respectively (Additional file 2: Figure S1b, Fig. 1b). The sequential TUNEL assay revealed that the TUNEL-positive reaction was significantly elevated in B50-SHED, especially on day 3, compared to B0-SHED and B10-SHED (Additional file 2: Figure S1c, Fig. 1c). Flow cytometric analysis demonstrated that B50-SHED exhibited a markedly increased Annexin-V and 7AAD double-positive population compared to B0-SHED and B10-SHED 3 days after serum-depletion (Additional file 2: Figure S1d, Fig. 1d). These findings suggested that 50 μM unconjugated bilirubin has a pro-apoptotic effect on SHED, but 10 μM unconjugated bilirubin does not have this effect.

### Pamidronate suppresses bilirubin-induced apoptosis in SHED

**Fig. 2:**
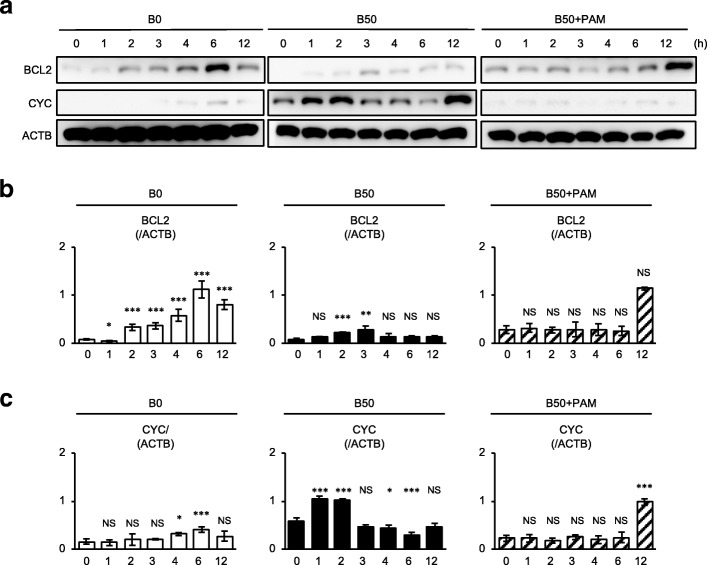
Pamidronate restores the inhibited BCL2 and increased cytochrome c in bilirubin-impaired SHED. SHED were cultured as described in Fig. 1a. **a**–**c** Sequential expression of BCL2 and cytochrome c (CYC) was analyzed at the indicated time by western blot analysis. Representative images of western blotting were shown (**a**). Results were shown as the representative expression of BCL2 to beta-actin (ACTB) (**b**) and CYC to ACTB (**c**) at each time point in each group. **a**–**c** B0, SHED treated with 0 μM bilirubin; B50, SHED treated with 50 μM bilirubin; B50+PAM, SHED treated with 50 μM bilirubin and 10 μM pamidronate. **b**, **c** *n* = 5 for all groups. Statistical analysis was performed as described in the “Methods” section. Graph bars showed the means ± SEM. *P < 0.05, **P < 0.01, and ***P < 0.005 (vs. 0 h in each group). NS, no significance

Given the present findings regarding cell death of SHED, the effects of pamidronate (10 μM) on cell death were examined under the high concentration (50 μM) unconjugated bilirubin in serum-depleted condition (Fig. 1a). The cell viability assay revealed that pamidronate treatment improved the suppressed viability of B50-SHED (Fig. 2b). TUNEL staining and flow cytometric analysis showed that pamidronate treatment reduced the bilirubin-induced cell death of B50-SHED (Fig. 2c, d).

**Fig. 3:**
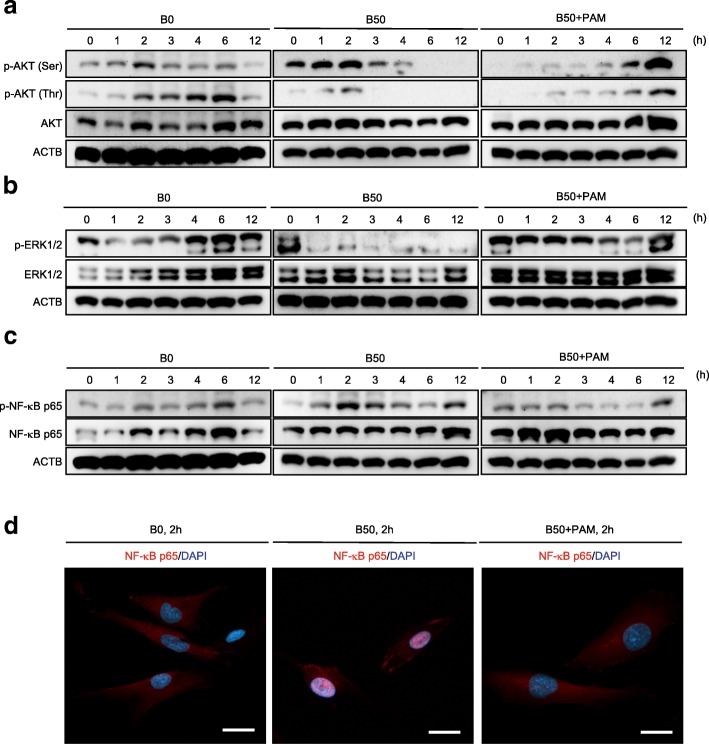
Pamidronate restores the altered phosphorylation of AKT, ERK1/2, and NF-κB p65 in bilirubin-impaired SHED. SHED were cultured as described in Fig. 1a. **a**–**c** Sequential expression of AKT, ERK1/2, and NF-κB p65 and their phosphoproteins, p-AKT (Ser), p-AKT (Thr), p-ERK1/2, and p-NF-κB p65, was analyzed at the indicated time by western blot analysis. Representative images of western blotting were shown. **d** SHED were stimulated with 0 μM bilirubin (B0), 50 μM bilirubin (B50), and 50 μM bilirubin plus 10 μM pamidronate (B50+PAM) under serum-depleted condition for 2 h. Nuclear translocation of NF-κB p65 in SHED by immunofluorescent microscopy. Nucleus was stained with 4′,6-diamidino-2-phenylindole (DAPI). Representative images of the localization of NF-κB p65 in SHED after 2 h of the stimulation. Bars = 20 μm. **a**–**c** *n* = 5 for all groups. Statistical analysis was performed as described in the “Methods” section. Graph bars showed the means ± SEM. *P < 0.05, **P < 0.01, and ***P < 0.005 (vs. 0 h in each group). NS, no significance

**Fig. 4:**
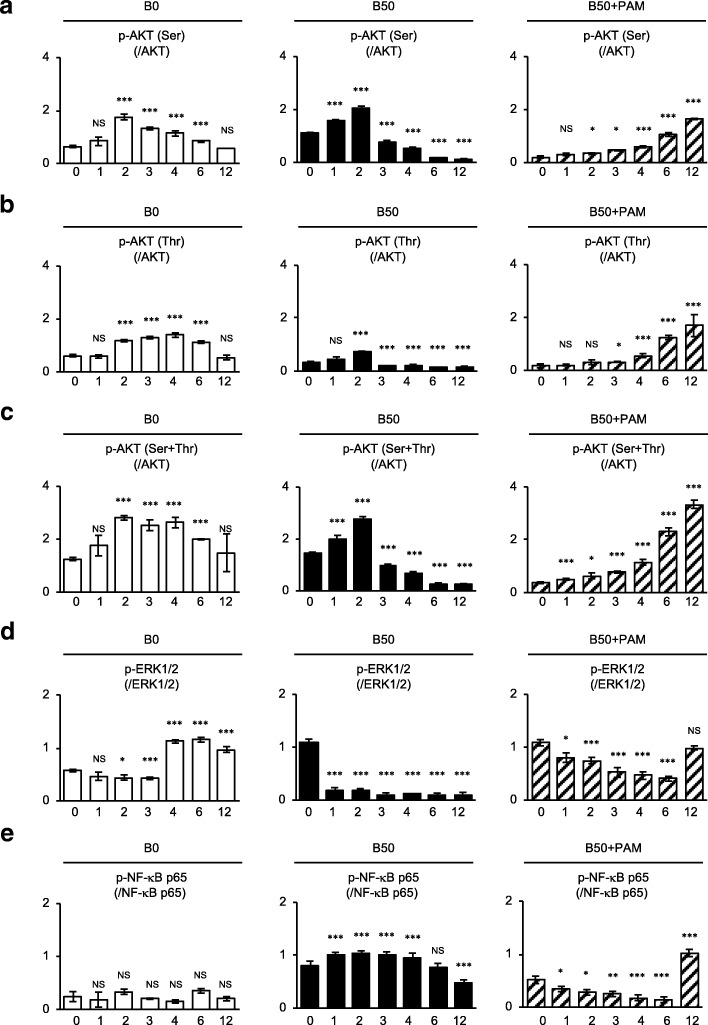
Effects of pamidronate on the kinetics of phosphorylated AKT, ERK1/2, and NF-κB p65 in bilirubin-impaired SHED. The results by western blot analysis were shown in Fig. 3. Relative phosphorylated expression of p-AKT (Ser) to AKT (**a**), p-AKT (Thr) to AKT (**b**), p-AKT (Ser) and p-AKT (Thr) [p-AKT (Ser+Thr)] to AKT (**c**), p-ERK1/2 to ERK1/2 (**d**), and p-NF-κB p65 to NF-κB p65 (**e**) was analyzed at each time point in each group. **a**–**e** B0, SHED treated with 0 μM bilirubin; B50, SHED treated with 50 μM bilirubin; B50+PAM, SHED treated with 50 μM bilirubin and 10 μM pamidronate. *n* = 5 for all groups. Statistical analysis was performed as described in the “Methods” section. Graph bars showed the means ± SEM. *P < 0.05 and ***P < 0.005. (vs. 0 h in each group). NS, no significance

To understand the mechanisms of the bilirubin-induced SHED death and the pamidronate-mediated rescue, we investigated the sequential expression of caspase 3, BCL2, and cytochrome c, which participate in mitochondria-mediated apoptosis. The western blot analysis showed that B50-SHED expressed the cleaved caspase 3 associated with the suppression of BCL2 and enhancement of cytochrome c in comparison with B0-SHED (Figs. 2e–h and 3, Additional file 2: Figures S2 and S3). Of interest, pamidronate treatment improved the bilirubin-exerted mitochondria-mediated apoptosis in B50-SHED (Figs. 2e–h and 3, Additional file 2: Figures S2 and S3). According to our current study [13], the suppressed phosphorylation of AKT and ERK1/2 and the enhanced phosphorylation of NF-κB were induced in the pro-apoptotic process in bilirubin-stimulated SHED (Figs. 3 and 4, Additional file 2: Figures S4 and S5). The present sequential analysis showed that pamidronate treatment improved the bilirubin-induced alternation of phosphorylation of the AKT, ERK1/2, and NF-κB p65 signal pathways in B50-SHED (Figs. 3a–c and 4, Additional file 2: Figures S4 and S5). Further immunofluorescent analysis confirmed that pamidronate treatment markedly canceled the nuclear translocation of NF-κB p65 in B50-SHED (Fig. 3d), as seen in SHED stimulated with TNFα (Additional file 2: Figure S6), suggesting that pamidronate treatment inhibited the enhanced NF-κB p65-mediated gene regulation in bilirubin-impaired SHED.

### Pamidronate rescues bilirubin-impaired in vitro dentinogenic function in SHED

**Fig. 5:**
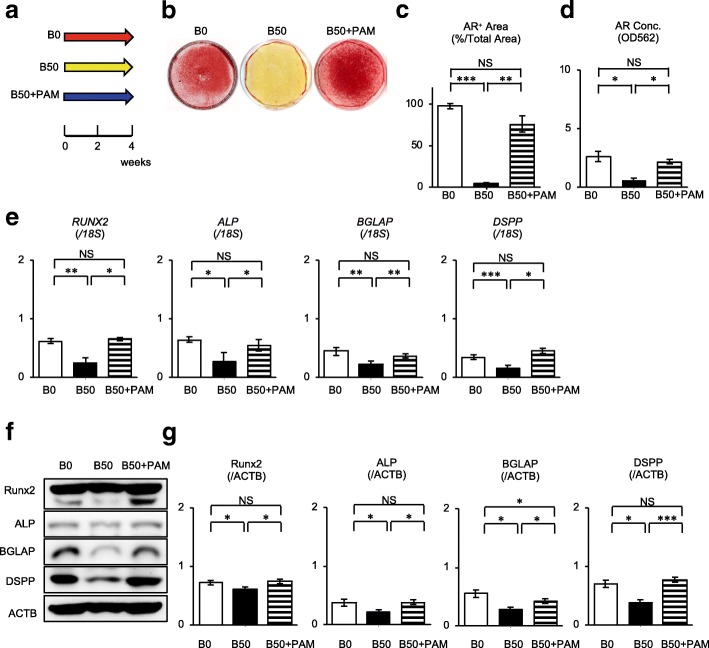
Pamidronate rescues the bilirubin-impaired dentinogenic capacity of SHED. **a** A schema of pamidronate treatment in bilirubin-impaired SHED under dentinogenic condition. SHED were cultured under dentinogenic condition stimulated with 0 μM bilirubin (B0), 50 μM bilirubin (B50), and 50 μM bilirubin plus 10 μM pamidronate (B50+PAM). **b**–**d** Calcium deposition assay after 4 weeks of the dentinogenic induction was performed by alizarin red (AR) staining. Representative images of AR staining were shown (**b**). AR-positive (AR^+^) area (**c**) and AR contents (**d**) in the cultures were measured. **e**–**g** Expression of odontoblast-specific genes and proteins after 2 weeks of the dentinogenic induction was analyzed. Relative expression of runt-related transcription factor 2 (*RUNX2*), alkaline phosphatase (*ALP*), bone gamma-carboxyglutamic acid-containing protein (*BGLAP*), and dentin sialophosphoprotein (*DSPP*) genes to 18S rRNA (*18S*) was shown by real-time RT-PCR (**e**). Representative western blotting images of RUNX2, ALP, BGLAP, and DSPP were shown (**f**). Relative expression of RUNX2, ALP, BGLAP, and DSPP to beta-actin (ACTB) was analyzed (**g**). **c**–**e**, **g** *n* = 5 for all groups. Statistical analysis was performed as described in the “Methods” section. Graph bars showed the means ± SEM. *P < 0.05, **P < 0.01, and ***P < 0.005. NS, no significance

Our recent study demonstrated that 50 μM unconjugated bilirubin damaged the dentinogenic capacity of SHED [13]. Given the present findings that pamidronate suppresses bilirubin-induced apoptosis, we examined the effects of pamidronate on the in vitro mineralized tissue-forming capacity of SHED under 50-μM unconjugated bilirubin exposure (Fig. 5a). Alizarin red staining showed that pamidronate treatment improved the impaired dentinogenic capacity of B50-SHED (Fig. 5b–d). Real-time RT-PCR and western blot assays demonstrated that pamidronate treatment recovered the suppressed expression of genes and proteins for runt-related transcription factor 2 (RUNX2), alkaline phosphatase (ALP), bone gamma-carboxyglutamic acid-containing protein (BGLAP), and dentin sialophosphoprotein (DSPP) in B50-SHED 2 weeks after the dentinogenic induction (Fig. 5e–g) (Additional file 1).

We then analyzed the effects of pamidronate on the expression of AKT, ERK1/2, and NF-κB and the phosphorylation of AKT, ERK1/2, and NF-κB in the in vitro dentinogenic differentiation in SHED under 50-μM unconjugated bilirubin exposure after 2 weeks of the odontogenic induction by western blot analysis. According to our current study [13], 50 μM unconjugated bilirubin suppressed phosphorylation of AKT and ERK1/2 and the enhanced phosphorylation of NF-κB were induced in bilirubin-stimulated SHED under odontogenic condition (Additional file 2: Figure S7). Western blot analysis showed that pamidronate treatment restored the altered phosphorylation of AKT, ERK1/2, and NF-κB p65 in bilirubin-stimulated SHED under odontogenic condition after 2 weeks of the odontogenic induction (Additional file 2:Figure S7).

### Pamidronate restores the capacity of SHED for mineralized tissue formation in vivo following bilirubin-induced impairment

**Fig. 6:**
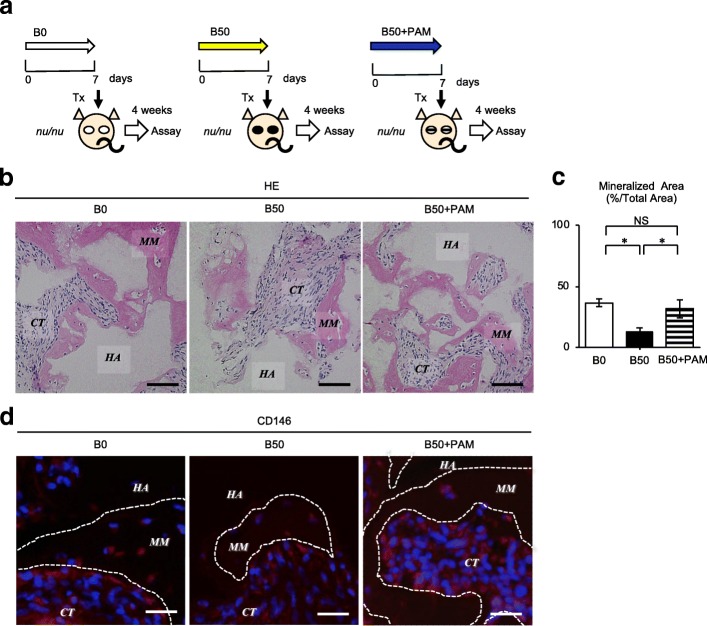
Pamidronate restores in vivo mineralized tissue-forming capacity in bilirubin-impaired SHED. **a** Schemata of SHED cultures and subcutaneous transplantation (Tx) into immunocompromised Balb/c *nu/nu* mice (*nu/nu*). SHED were precultured with 0 μM bilirubin (B0), 50 μM bilirubin (B50), and 50 μM bilirubin plus 10 μM pamidronate (B50+PAM) and were subcutaneously transplanted with hydroxyapatite/tricalcium phosphate particles (HA/TCP) in immunocompromised mice. **b**–**d** Histological analysis was performed after 4 weeks of the transplantation. Representative transplant images by hematoxylin and eosin staining (HE) were shown (**b**). De novo mineralized tissue area in SHED transplants was measured as described in the “Methods” section. *n* = 5 for all groups. Statistical analysis was performed as described in the “Methods” section. Graph bars showed the means ± SD. *P < 0.05. NS, no significance (**c**). Representative transplant images by immunofluorescence with anti-human CD146 antibody were shown. White-dot circled area: newly formed mineralized area (**d**). **b**, **d** CT, connective tissue; HA, HA/TCP; MM, mineralized tissue. Bar = 100 μm

We examined the effects of pamidronate on the in vivo mineralized tissue-forming capacity of bilirubin-exposed SHED (Fig. 6a). Histological analysis showed that the in vivo mineralized tissue-forming capacity was reduced by 50-μM unconjugated bilirubin treatment. Meanwhile, the bilirubin-impaired in vivo mineralized tissue-forming capacity of B50-SHED was markedly improved by pamidronate treatment (Fig. 6b, c). Immunofluorescence analysis demonstrated that human CD146-positive cells were lining the newly formed mineralized matrix in each transplant group (Fig. 6d), suggesting that SHED conditioned with unconjugated bilirubin and pamidronate reflected, at least in partially, the formation rate of de novo mineralized tissues formed in the present subcutaneous transplant system.

## Discussion

Although our study demonstrates that 50 μM unconjugated bilirubin causes cell death in SHED, the concentration of the unconjugated bilirubin and the underlying mechanisms have not been fully elucidated. Unconjugated bilirubin behaves as both an anti-oxidant at normal physiological concentrations and a pro-oxidant at high concentrations [24, 25]. A high concentration of unconjugated bilirubin acts as a pro-oxidant to generate reactive oxygen species (ROS), resulting in DNA strand cleavage associated with mitochondrial apoptosis and cell dysfunction [26–28]. Unconjugated bilirubin suppresses the AKT and ERK1/2 signal pathways and enhances the NF-κB signal pathway involved in apoptosis in neural cells [29–31]. Given the present findings by the analysis of sequential intracellular signal pathways in SHED after exposure to different concentrations of unconjugated bilirubin, 50 μM unconjugated bilirubin is suggested as having a pro-apoptotic effect on the mitochondrial BCL2-cytochrome c-caspase 3 pathway via the inhibition of AKT and ERK1/2 and the activation of NF-κB. Further studies may elucidate the participation of oxidative stress including ROS on the pro-apoptotic process in SHED exposed to unconjugated bilirubin.

The change in total blood and conjugated bilirubin concentrations in patients with BA leads to an increase in the blood concentration of unconjugated bilirubin [32]. Given that tooth injury develops under a concentration of more than approximately 3 mg/dL (50 μM) of total blood bilirubin in BA patients [33], the present and recent [13] findings regarding in vivo and in vitro dentinogenic dysfunction in bilirubin-stimulated SHED are considerable and supported by the present mitochondria-mediated apoptosis in bilirubin-stimulated SHED. Previous studies using unconjugated bilirubin and hyperbilirubinemia patient-derived serum support that bilirubin-mediated apoptosis and osteogenic dysfunction of human osteoblasts are associated with bone reduction in hyperbilirubinemia [7, 8]. Therefore, these findings suggest pediatric hyperbilirubinemia causes clinical dentin hypoplasia of deciduous teeth through excess bilirubin-stimulated apoptosis in SHED.

Our recent ex vivo interferon-γ treatment with irreversible pulpitis-damaged dentinogenic stem cells suggested that a pharmacological approach has potential to recover the pathologically impaired functions of patient-derived dental pulp stem cells [34]. Because of the present bilirubin-impaired SHED dysfunction, the hyperbilirubinemia in pediatric patients with BA waiting for liver transplantation deteriorates the clinical dental condition and is associated with hypodontia. However, no form of dental care has managed to prevent dental failure under hyperbilirubinemia. Previous reports indicate the anti-apoptotic effects of bisphosphonate on osteocytes and osteoblasts [35, 36]. Alendronate exhibits anti-apoptotic effects via the activation of the ERK pathway and inactivation of pro-apoptotic mitochondrial BAD [37]. The anti-apoptotic effects of pamidronate require the activation of AKT and ERK1/2 and suppression of NF-kB [35, 38–40]. Given the present pamidronate-mediated rescuing of bilirubin-impaired mitochondrial apoptosis in SHED associated with AKT, ERK1/2, and suppressed NF-kB signal pathways, pamidronate treatment in pediatric patients with hyperbilirubinemia might prevent dentin hypoplasia. Indeed, pamidronate is used as safety medicine to reduce bone fracture in pediatric patients with osteogenesis imperfecta (OI) [41]. OI patients with pamidronate treatment show a delaying dental eruption in comparison with OI patients with naïve treatment, but not indistinguishable from normal ones [42]. Further studies will be necessary to understand the effects of pamidronate alone on cell functions including cell apoptosis and odontogenesis and the related intracellular signal transduction in SHED for pediatric patients receiving pamidronate.

## Conclusion

The present findings suggest that tooth abnormalities in patients with hyperbilirubinemia might be due to bilirubin-induced cell death and dentinogenic dysfunction in dentinogenic stem cells via altered AKT, ERK1/2, and NF-κB signal pathways. The bilirubin-induced impairments in dentinogenic stem cells were successfully reversed by pamidronate treatment. This study proposes a new experimental model to elucidate disease mechanisms using the stem cells implicated in the disease. Moreover, the present study might also provide a novel endogenous stem cell-targeting pharmaceutical therapy to cure or prevent disorders associated with damaged stem cells in the future.

## Additional files

Additional file 1: Supplementary Methods. (DOCX 22 kb)

Additional file 2: Supplementary figures. (ZIP 676 kb)

## Abbreviations

ALP: Alkaline phosphatase
B0-SHED: 0-μM unconjugated bilirubin-treated SHED
B10-SHED: 10-μM unconjugated bilirubin-treated SHED
B50-SHED: 50-μM unconjugated bilirubin-treated SHED
BA: Biliary atresia
BGLAP: Bone gamma-carboxyglutamic acid-containing protein
DSPP: Dentin sialophosphoprotein
ERK1/2: Extracellular-regulated kinases 1/2
MSCs: Mesenchymal stem cells
NF-κB: Nuclear factor kappa B
ROS: Reactive oxygen species
RUNX2: Runt-related transcription factor 2
SEM: Standard error of mean
SHED: Stem cells from human exfoliated deciduous teeth
TNFα: Tumor necrosis factor alpha
TUNEL: TdT-mediated dUTP nick end labeling
