# Supplementary material for: Pamidronate decreases bilirubin-impaired cell death and improves dentinogenic dysfunction of stem cells from human deciduous teeth
Source: Stem Cell Res Ther. 2018 Nov 8;9:303. doi: 10.1186/s13287-018-1042-7 (PMC6225573; doi:10.1186/s13287-018-1042-7)
Supplement: Supplementary file 1 — Supplementary Methods. (DOCX 22 kb) [file 13287_2018_1042_MOESM1_ESM.docx]

**Supplementary Methods**

**Isolation and culture of stem cells from human deciduous teeth (SHED)**

The isolation and culture of stem cells from human deciduous teeth (SHED) were performed according to our previous reports (Yamaza et al., 2010; Ma et al., 2012). Dental pulp tissues of human deciduous teeth were digested with 0.3% collagenase type I (Worthington Biochemicals, Lakewood, NJ) and 0.4% dispase II (Godo Syusei, Tokyo, Japan) for 60 min at 37^o^C, and the obtained cells were seeded on culture flasks. After 3 hours, the cultures were washed with sterilized phosphate-buffered saline (PBS). The adherent cells were incubated with regular growth medium. The growth medium consisted of 15% fetal bovine serum (FBS) (Equitech-Bio, Kerrville, TX), 100 μM L-ascorbic acid 2-phosphate (Wako Pure Chemicals, Osaka, Japan), 2 mM L-glutamine (Nacalai Tesque, Kyoto, Japan), 100 U/ml penicillin and 100 μg/ml streptomycin (Nacalai Tesque) in alpha Modification of Eagle's Medium (α-MEM; Invitrogen, Carlsbad, CA). The isolated cells showed the capacity to form attached colonies consisting of spindle-shaped cells on plastic culture dishes (**data not shown**). The adherent colony-forming cells were passaged and sub-cultured. The medium was changed twice a week. To confirm the criteria of our isolated cells as MSCs, the passage 3 (P3) cells were stained with R-phycoerythrin-conjugated anti-human CD146, CD105, CD90, CD73, CD34, CD45 and CD14 antibodies (**data not shown**)) and assessed by BD FACSVerse™ flow cytometer (BD Bioscience, Franklin Lake, NJ) as described previously (Yamaza et al., 2010). The cells expressed positive to CD146, CD73, CD105 and CD90, but negative to hematopoietic markers (CD34, CD45 and CD14) (**data not shown**). The specific antibodies used in the present flow cytometry were summarized in the **Supplementary Table 1**. The P3 cells were also cultured under dentinogenic/osteogenic, chondrogenic and adipogenic conditions as described previously (Ma et al., 2012). The induced SHED exhibited multi-differentiation capacity into 3types of classical mesenchymal lineage cells including odontoblasts/osteoblasts, chondrocytes, and adipocytes (**data not shown**). These phenotypes indicated that our isolated SHED fulfilled the minimal and standard criteria for MSCs (Dominici et al., 2006). Therefore, the P3 cells were used as SHED for further experiments in this study.

**Preparation of bilirubin solutions**

The bilirubin dilution was according to the previous report (Trost et al., 1993). Briefly, stock solution of unconjugated bilirubin (1600 μM) (Sigma-Aldrich, St. Louis, Mo) was dissolved into 0.1 N NaOH under shield and adjusted to pH 7.2-7.4 with 0.1N HCl followed by sterilizing through a filter (0.22 μm pore size). Normal blood concentration of bilirubin is below 1.2 [mg](https://en.wikipedia.org/wiki/Milligram)/[dL](https://en.wikipedia.org/wiki/Decilitre) (<25 μM). Blood total bilirubin concentration higher than approximately 3 mg/dL (>50 μM) leads to obvious green staining in deciduous teeth, as well as jaundice (Zaia et al., 1993). In this *in vitro* experiment, we used 3 types of the bilirubin working concentration, 0, 10 or 50 μM. The stock solution was diluted with albumin (Sigma-Aldrich) (molar ratio of bilirubin to albumin of 1.5:1.0) to the working concentration of the bilirubin (10 and 50 μM). Control cultures were treated with the same amount of NaOH and albumin without bilirubin (0 μM).

**Real-time reverse transcript-polymerase chain reaction (RT-PCR) assay**

To isolate total RNA from the cells, culture samples were treated with TRIzol (Invitrogen), digested with DNase I (Promega, Madison, WI), and purified using an RNeasy Mini Kit (Qiagen, [Venlo](http://en.wikipedia.org/wiki/Venlo), [Netherlands](http://en.wikipedia.org/wiki/Netherlands)). One microgram (1µg) of purified total RNA was reverse-transcribed with Revertra Ace qPCR kit (TOYOBO, Osaka, Japan) for real-time reverse transcript-polymerase chain reaction (RT-PCR). Real-time RT-PCR was subsequently performed using TaqMan Gene Expression Master Mix (Applied Biosystems, Foster City, CA) and targeted TaqMan probes with a Light Cycler 96 real-time PCR system (Roche, Indianapolis, IN). 18S ribosomal RNA was used as the internal control. All probes were purchased form Applied Biosystems and summarized in the **Supplementary Table 2**. The expression of each targeted gene was normalized with 18S ribosomal RNA expression.

**Western blot analysis**

Cultured samples were collected at the corresponding period. All samples were lysed in M-PER mammalian protein extraction reagent (Thermo Fisher Scientific, Rockford, IL) supplemented with proteinase inhibitor cocktail (Nacalai Tesque) and phosphatase inhibitor PhoSTOP (Roche, Basel, Schweizerische). They were separated by TGX FastCast acrylamide gels (Bio Rad Laboratories, Hercules, CA) and transferred on a Trans-Blot Turbo transfer system (Bio Rad Laboratories). The membranes were blocked with 5% skimmed milk in Tris-buffered saline (150 mM NaCl and 20 mM Tris–HCl, pH 7.2) at room temperature for 1 h and then incubated with primary antibodies at 4^o^C overnight. The primary antibodies used in this study were summarized in the **Supplementary Table 3**. They were treated with horseradish peroxidase-conjugated donkey anti-rabbit secondary antibody (1:1000; Santa Cruz Biotechnology) at room temperature for 1 h. Each membrane was treated with WB stripping solution strong (Nacalai) to reprobe primary and secondary antibodies and stained with anti-β-actin antibody (Sigma-Aldrich), followed by incubating with horseradish peroxidase-conjugated donkey anti-mouse IgG secondary antibody (1:1000; Santa Cruz Biotechnology). The bound antibodies were visualized using SuperSignal West Pico (Thermo Fisher Scientific) on an ImageQuant LAS 4010 imager (GE Healthcare Life Science, Pittsburgh, PA). The intensity of each band was measured by using Image-J (National Institutes of Health, Bethesda, MD) and normalized with the intensity of the corresponding β-actin band as the internal control.

**Supplementary References**

Dominici M, Le Blanc K, Mueller I, Slaper-Cortenbach I, Marini F. 2006. Minimal criteria for defining multipotent mesenchymal stromal cells. The International Society for Cellular Therapy position statement. Cytotherapy 8;315-317.

Ma L, Makino Y, Yamaza H, Akiyama K, Hoshino Y, Song G, Kukita T, Nonaka K, Shi S, Yamaza T. 2012. Cryopreserved dental pulp tissues of exfoliated deciduous teeth is a feasible stem cell resource for regenerative medicine. PLoS One 7;e51777.

Trost GR, Nagatani K, Goknur AB, Haworth RA, Odell GB, Duff TA. 1993. Bilirubin levels in subarachnoid clot and effects on canine arterial smooth muscle cells. Stroke 24;1241-1245.

Yamaza T, Kentaro A, Chen C, Liu Y, Shi Y, Gronthos S, Wang S, Shi S. 2010. Immunomodulatory properties of stem cells from human exfoliated deciduous teeth. Stem Cell Res Ther 1;5.

Zaia AA, Graner E, de Almeida OP, Scully C. 1993. Oral changes associated with BA and liver transplantation. J Clin Pediatr Dent 181;38-42.

**Supplementary Table 1.** List of antibodies for flow cytometry.

**Names of antibodies Types of antibodies Names of Suppliers**

anti-CD14 R-PE -conjugated eBioscience (San Diego, CA)

anti-CD34 R-PE-conjugated eBioscience (San Diego, CA)

anti-CD45 R-PE-conjugated eBioscience (San Diego, CA)

anti-CD73 R-PE-conjugated eBioscience (San Diego, CA)

anti-CD90 R-PE-conjugated eBioscience (San Diego, CA)

anti-CD105 R-PE-conjugated eBioscience (San Diego, CA)

anti-CD146 R-PE-conjugated eBioscience (San Diego, CA)

R-PE: R-phycoerythrin

**Supplementary Table 2.** List of TaqMan probes for real-time RT-PCR

**Names of genes Gene assay ID Numbers**

#### *ALP* Hs01029144_m1

#### *BGLAP* Hs01587814_g1

#### *DSPP* Hs00171962_m1

*RUNX2* Hs00231692_m1

Ribosomal RNA, 18S Hs99999901_s1

**Supplementary Table 3.** List of specific antibodies for western blotting and immunofluorescence.

**Names of antibodies Types of antibodies Suppliers**

anti-ACTB purified Merck (Darmstadt, Germany)

anti-AKT purified Cell Signaling Technology (Danvers, MA)

anti-AKT (Ser473), phosphorylated purified Cell Signaling Technology (Danvers, MA)

anti-BCL2 purified Cell Signaling Technology (Danvers, MA)

anti-CASP3 purified Cell Signaling Technology (Danvers, MA)

anti-CYC purified Cell Signaling Technology (Danvers, MA)

anti-CD146 purified Abcam (Cambridge, England)

anti-DSPP purified Santa Cruz Biotechnology (Dallas, TX)

anti-ERK1/2 purified Cell Signaling Technology (Danvers, MA)

anti-ERK1/2, phosphorylated purified Cell Signaling Technology (Danvers, MA)

anti-NF-κB p65 purified Cell Signaling Technology (Danvers, MA)

anti- NF-κB p65, phosphorylated purified Cell Signaling Technology (Danvers, MA)

anti-mitochondria, human purified Merck (Darmstadt, Germany)

anti-RUNX2 purified Abcam (Cambridge, England)

ACTB: actin, β

CASP3: caspase 3

CYC: cytochrome c

DSPP: dentin sialophosphoprotein

ERK: extracellular signal-regulated kinases

NF-kB: nuclear factor kappa B

RUNX2: runt-related transcription factor 2
