# Supplementary material for: Pamidronate decreases bilirubin-impaired cell death and improves dentinogenic dysfunction of stem cells from human deciduous teeth
Source: Stem Cell Res Ther. 2018 Nov 8;9:303. doi: 10.1186/s13287-018-1042-7 (PMC6225573; doi:10.1186/s13287-018-1042-7)
Supplement: Supplementary file 2 — Supplementary figures. (ZIP 676 kb) [file 13287_2018_1042_MOESM2_ESM.zip › Figure S1.pdf]

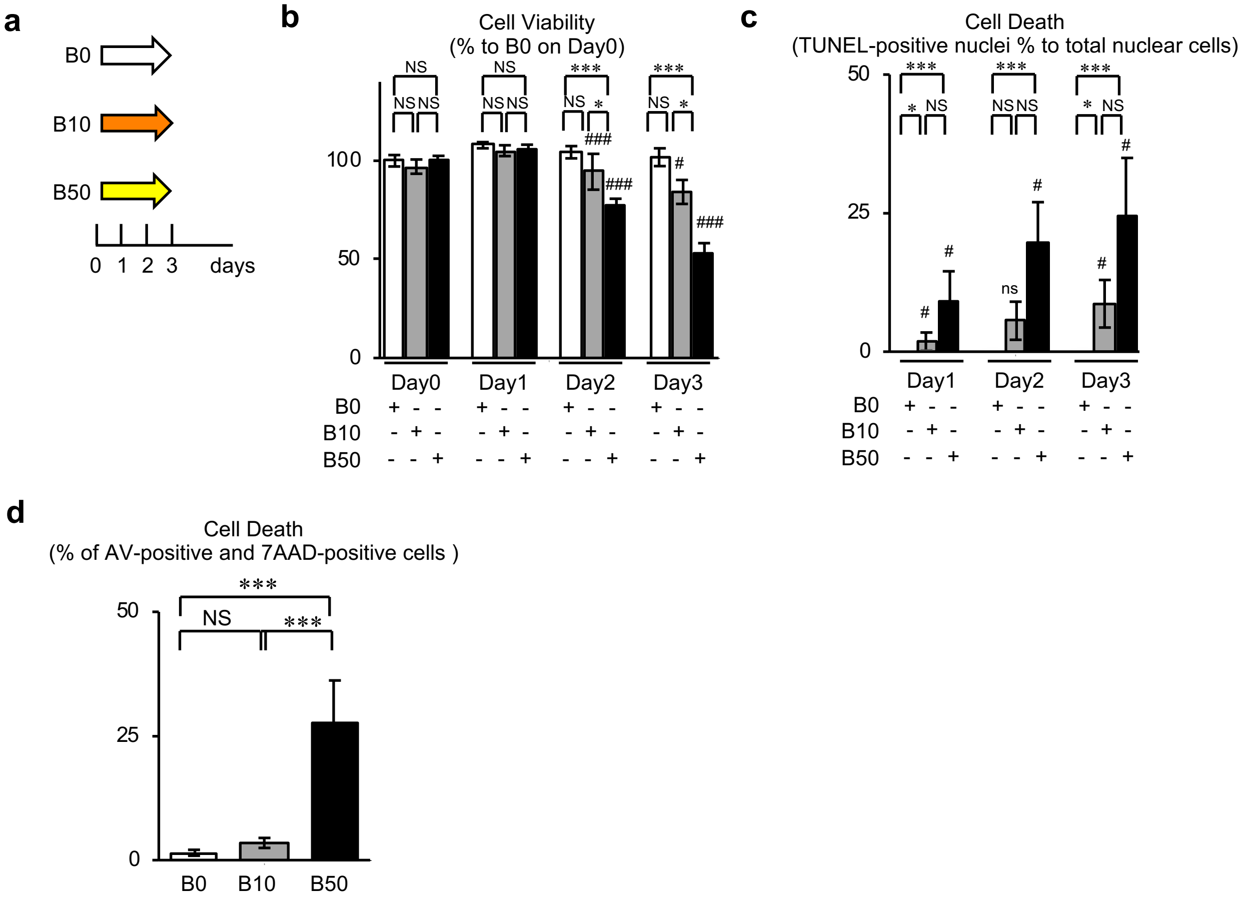

Supplementary Figure 1. Bilirubin induces cell death of SHED. (**a**) A scheme of SHED cultures for the cell proliferation and cell death assays. SHED were incubated with bilirubin (0, 10, and 50 μM) for 3 days under serum-depleted conditions. (**b**) Cell viability assay. Results were shown as a ratio (%) to the cell viability in B0 on Day 0. (**c**) TUNEL staining assay. Results were shown as a ratio (%) of TUNEL-positive nuclei to to total nuclear cells. (**d**) Flow cytometry with Annexin-V (AV) and 7AAD staining. Results were shown as a ratio (%) of AV-positive and &AAD-positive. **a**–**d**: B0: group treated with 0 μM bilirubin; B10: group treated with 10 μM bilirubin; B50: group treated with 50 μM bilirubin. **b**–**d**: n=5 for all groups. Statistical analysis was performed as described in the Methods. Graph bars showed the means ± SEM. $*P < 0.05$ and $***P < 0.005$. NS, no significance. **b, c**: $\#P < 0.05$ and $\#\#\#P < 0.005$ vs. B0 on Day 0. ns, no significance vs. B0 on Day 0.
