# Supplementary material for: Pamidronate decreases bilirubin-impaired cell death and improves dentinogenic dysfunction of stem cells from human deciduous teeth
Source: Stem Cell Res Ther. 2018 Nov 8;9:303. doi: 10.1186/s13287-018-1042-7 (PMC6225573; doi:10.1186/s13287-018-1042-7)
Supplement: Supplementary file 2 — Supplementary figures. (ZIP 676 kb) [file 13287_2018_1042_MOESM2_ESM.zip › Figure S2.pdf]

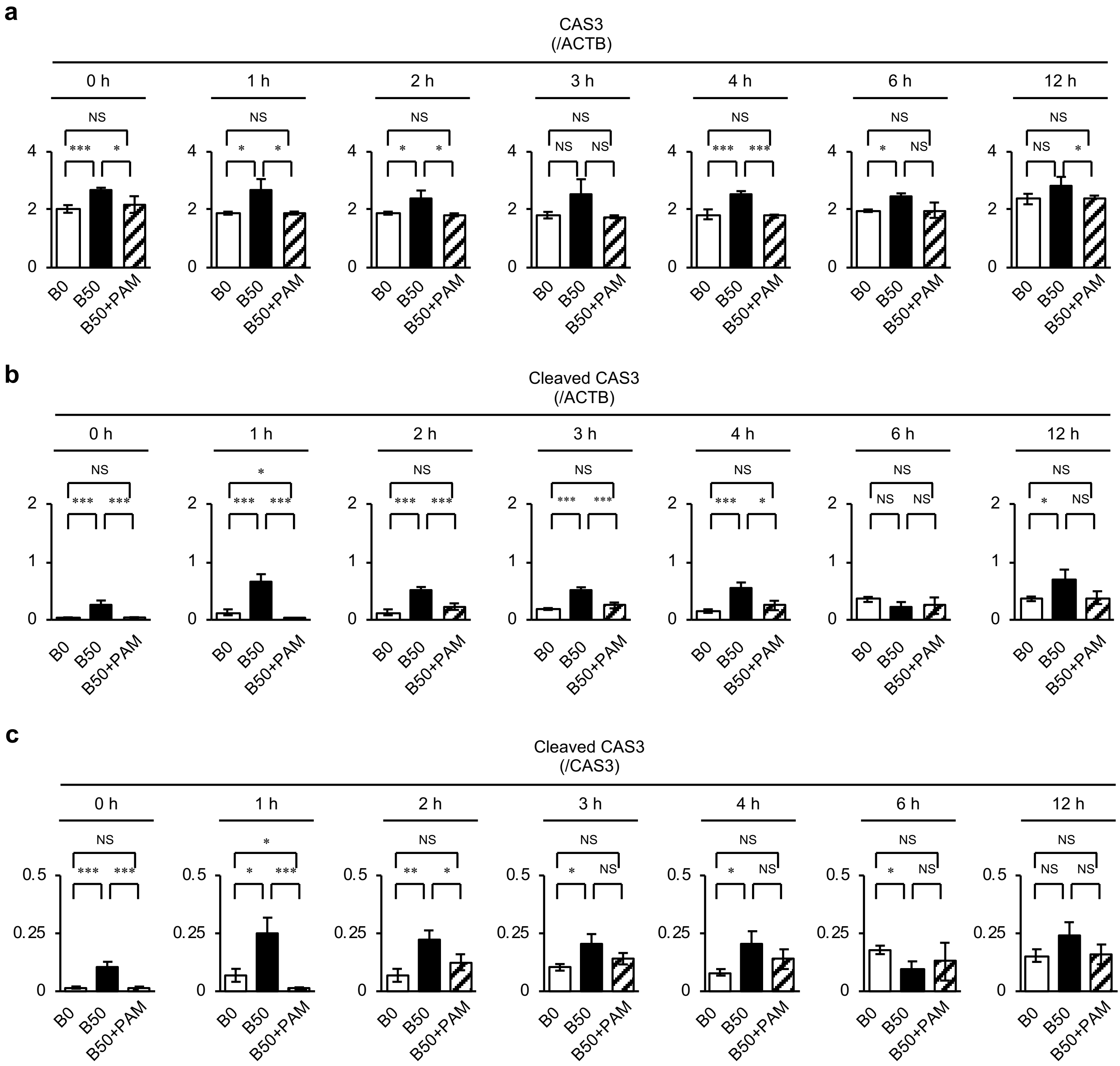

Supplementary Figure 2. Effects of pamidronate on the kinetics of caspase 3 and cleaved caspase 3 at each time point in bilirubin-impaired SHED. Sequential expression of caspase 3 (CASP3) and the cleaved caspase 3 (Cleaved CASP3) by western blot analysis were shown in Figure 1e. Results were shown as the relative expression of caspase 3 (CASP3) to beta-actin (ACTB) (**a**), cleaved caspase 3 (Cleaved CASP3) to beta-actin (**b**), and cleaved caspase 3 to caspase 3 (**c**) at each time point among the three groups. B0, SHED treated with 0 μM bilirubin; B50, SHED treated with 50 μM bilirubin; B50+PAM, SHED treated with 50 μM bilirubin and 10 μM pamidronate. n = 5 for all groups. Statistical analysis was performed as described in the Methods. Graph bars showed the means ± SEM. *P < 0.05, **P < 0.01, and ***P < 0.005. NS: no significance.
