# Supplementary material for: Pamidronate decreases bilirubin-impaired cell death and improves dentinogenic dysfunction of stem cells from human deciduous teeth
Source: Stem Cell Res Ther. 2018 Nov 8;9:303. doi: 10.1186/s13287-018-1042-7 (PMC6225573; doi:10.1186/s13287-018-1042-7)
Supplement: Supplementary file 2 — Supplementary figures. (ZIP 676 kb) [file 13287_2018_1042_MOESM2_ESM.zip › Figure S3.pdf]

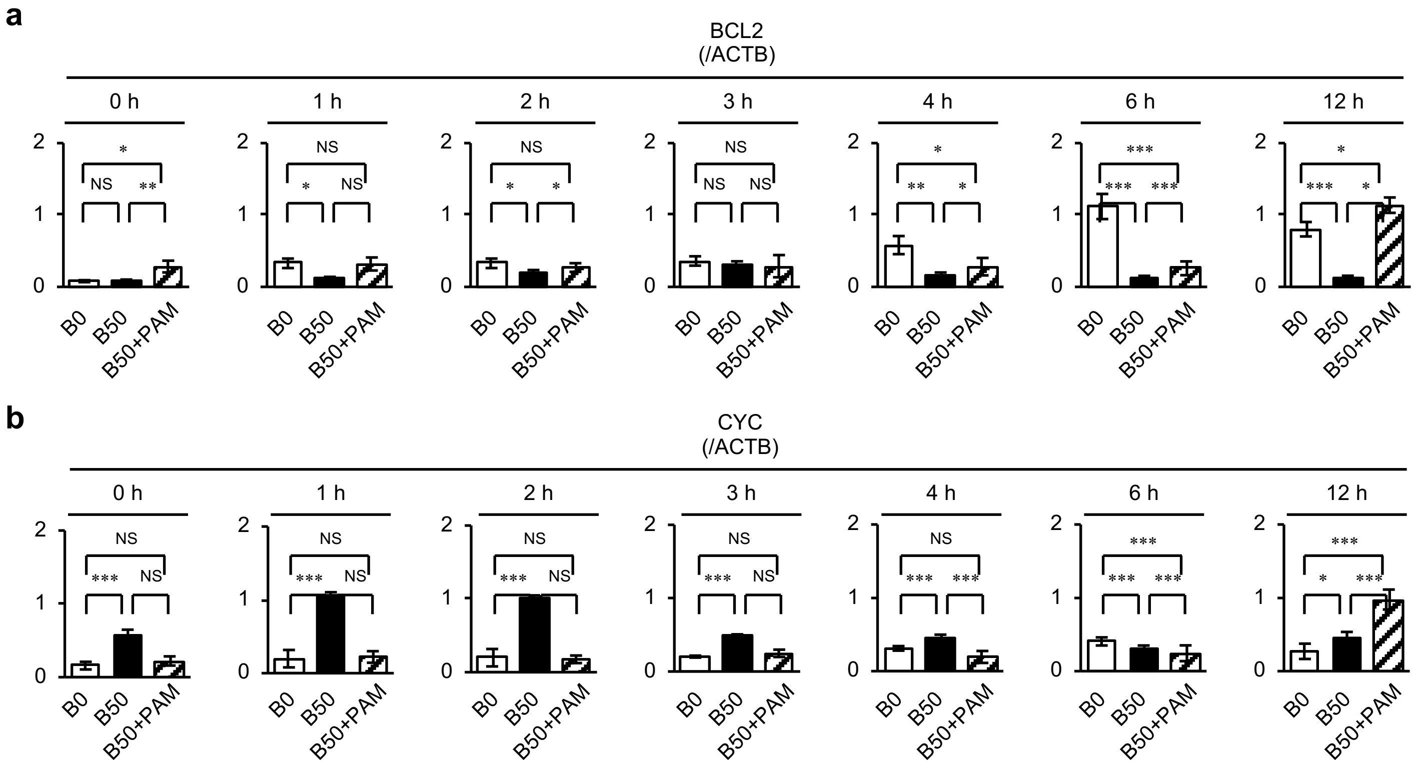

Supplementary Figure 3. Effects of pamidronate on the kinetics of BCL2 and cytochrome c at each time point in bilirubin-impaired SHED. Sequential expression of BCL2 and cytochrome c (CYC) by western blot analysis was shown in Figure 2a. Results were shown as the relative expression of BCL2/b-actin (BCL2/ACTB) (**a**) and cytochrome c/b-actin (CYC/ACTB) (**b**) at each time point among the three groups. B0, SHED treated with 0 μM bilirubin; B50, SHED treated with 50 μM bilirubin; B50+PAM, SHED treated with 50 μM bilirubin and 10 μM pamidronate. n = 5 for all groups. Statistical analysis was performed as described in the Methods. Graph bars showed the means ± SEM. *$P < 0.05$, **$P < 0.01$, and ***$P < 0.005$. NS: no significance.
