# Supplementary material for: Pamidronate decreases bilirubin-impaired cell death and improves dentinogenic dysfunction of stem cells from human deciduous teeth
Source: Stem Cell Res Ther. 2018 Nov 8;9:303. doi: 10.1186/s13287-018-1042-7 (PMC6225573; doi:10.1186/s13287-018-1042-7)
Supplement: Supplementary file 2 — Supplementary figures. (ZIP 676 kb) [file 13287_2018_1042_MOESM2_ESM.zip › Figure S4.pdf]

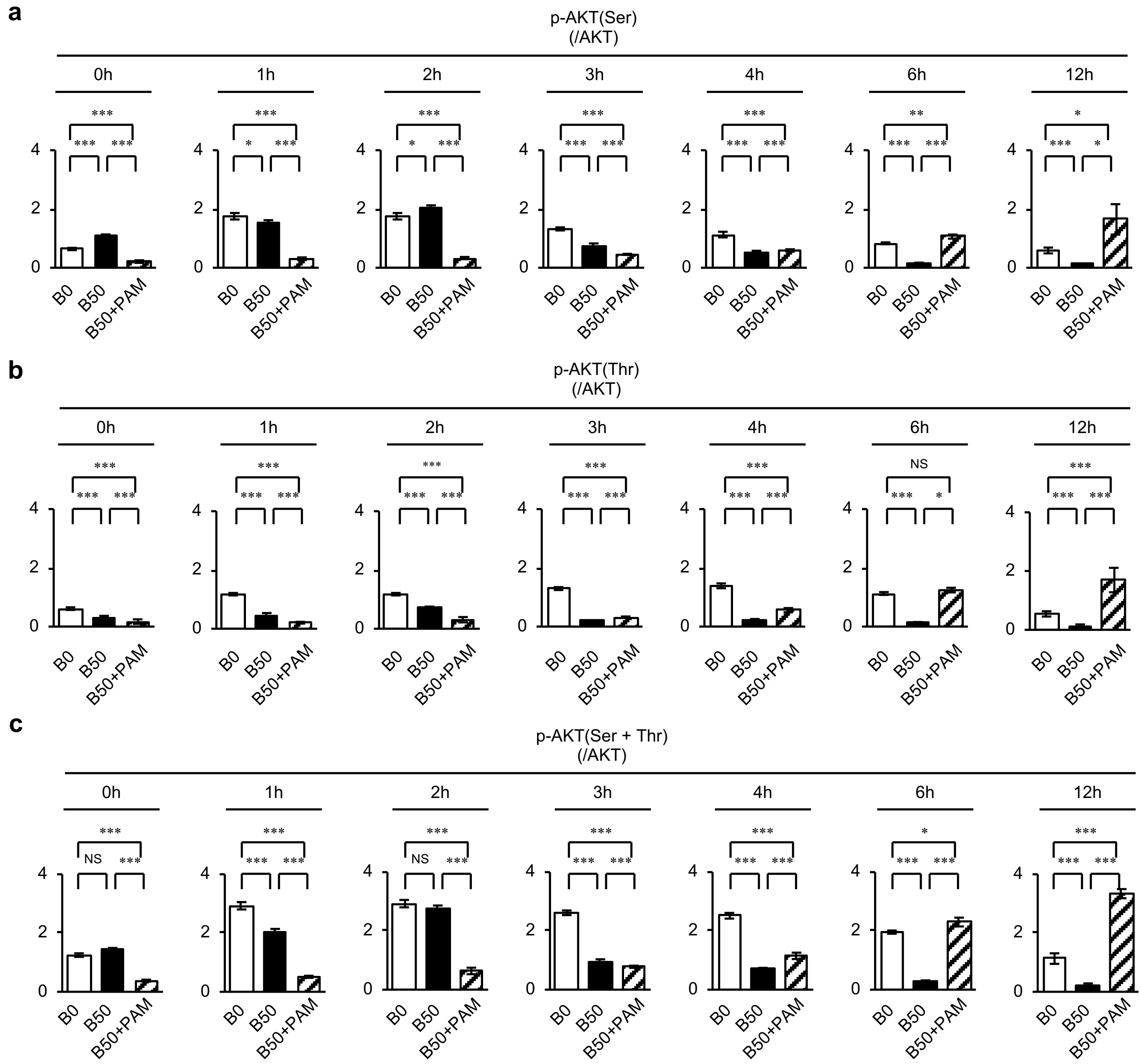

Supplementary Figure 4. Effects of pamidronate on the kinetics of phosphorylation of AKT at each time point in bilirubin-impaired SHED. Sequential expression of AKT and its phosphoprotein p-AKT (Ser) and p-AKT (Thr) by western blot analysis was shown in Figure 3a. Results were shown as the relative phosphorylated expression of p-AKT (Ser) (**a**), p-AKT (Thr) (**b**), and p-AKT (Ser) and p-AKT (Thr) [p-AKT (Ser) + p-AKT (Thr)] (**c**) to AKT, p-AKT (Ser)/AKT, p-AKT (Thr)/AKT, and p-AKT (Ser + Thr)/AKT, respectively, at each time point among the three groups. B0, SHED treated with 0 μM bilirubin; B50, SHED treated with 50 μM bilirubin; B50+PAM, SHED treated with 50 μM bilirubin and 10 μM pamidronate. n = 5 for all groups. Statistical analysis was performed as described in the Methods. Graph bars showed the means ± SEM. *$P < 0.05$, **$P < 0.01$ and ***$P < 0.005$. NS: no significance.
