# Supplementary material for: Pamidronate decreases bilirubin-impaired cell death and improves dentinogenic dysfunction of stem cells from human deciduous teeth
Source: Stem Cell Res Ther. 2018 Nov 8;9:303. doi: 10.1186/s13287-018-1042-7 (PMC6225573; doi:10.1186/s13287-018-1042-7)
Supplement: Supplementary file 2 — Supplementary figures. (ZIP 676 kb) [file 13287_2018_1042_MOESM2_ESM.zip › Figure S6.pdf]

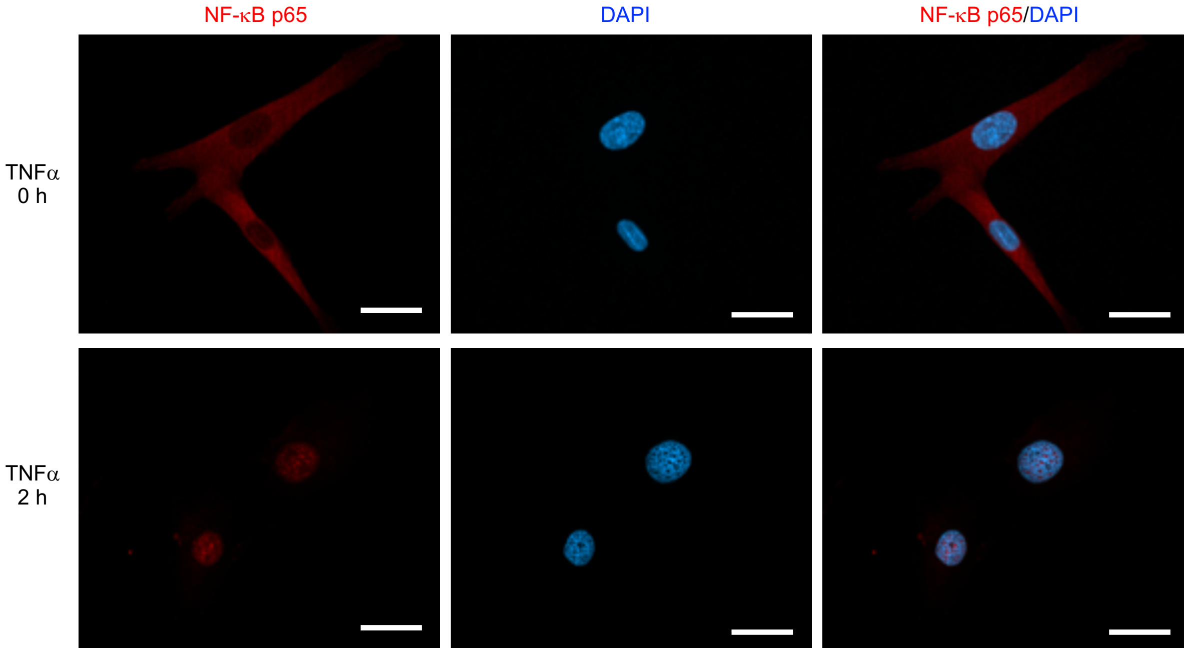

Supplementary Figure 6. Nuclear translocation of NF-kB p65 in SHED under tumor necrosis factor alpha (TNFα) stimulation. The cells were stimulated with TNFα (10 nM) under a serum-depleted condition for 2 h. Nuclear translocation of NF-κB p65 in SHED by immunofluorescent microscopy. Nucleus were stained with 4',6-diamidino-2-phenylindole (DAPI). Representative images of the localization of NF-κB p65 in SHED after 0 and 2 h of the stimulation. Bars = 20 μm.
