# Supplementary material for: Pamidronate decreases bilirubin-impaired cell death and improves dentinogenic dysfunction of stem cells from human deciduous teeth
Source: Stem Cell Res Ther. 2018 Nov 8;9:303. doi: 10.1186/s13287-018-1042-7 (PMC6225573; doi:10.1186/s13287-018-1042-7)
Supplement: Supplementary file 2 — Supplementary figures. (ZIP 676 kb) [file 13287_2018_1042_MOESM2_ESM.zip › Figure S7.pdf]

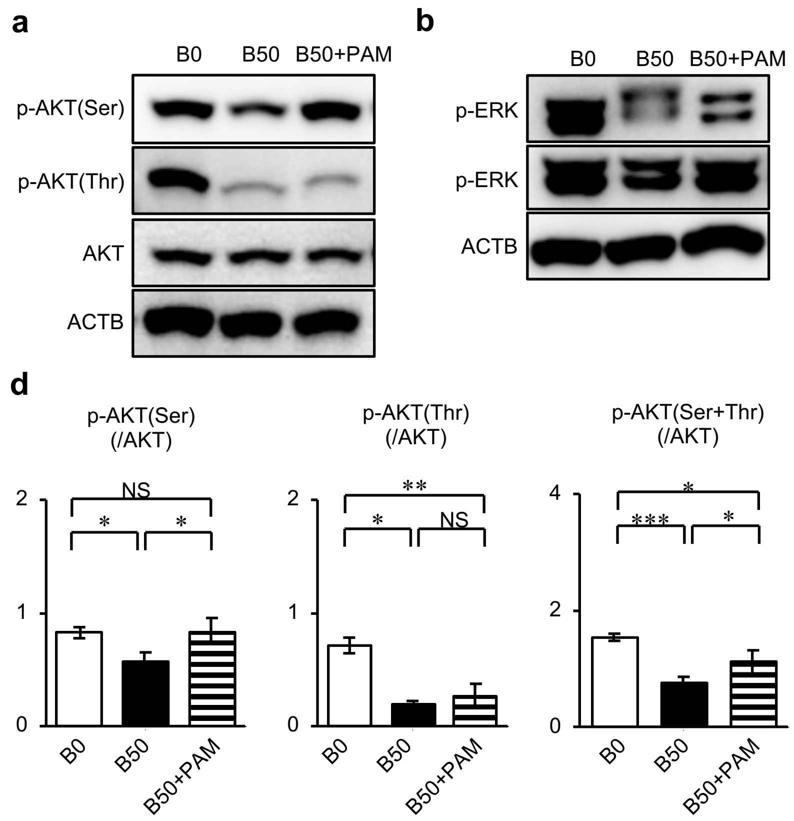

Supplementary Figure 7. Pamidronate restores the altered phosphorylation of AKT, ERK1/2, and NF-κB p65 in bilirubin-impaired SHED under dentinogenic condition. SHED were cultured as described in Figure 5a. Expression of AKT, ERK1/2, and NF-κB p65 and their phosphoproteins p-AKT (Ser), p-AKT (Thr), p-ERK1/2, and p-NF-κB p65 after 2 weeks of the dentinogenic induction was analyzed by western blotting. (**a-c**) Representative western blotting images are shown. (**d-f**) Results were shown as the relative phosphorylated expression of p-AKT (Ser) to AKT, p-AKT (Thr) to AKT, and p-AKT (Ser) and p-AKT (Thr) [p-AKT (Ser+Thr)] to AKT (**d**), p-ERK1/2 to ERK1/2 (**e**), and p-NF-κB p65 to NF-κB p65 (**f**). **a**–**f**: B0, SHED treated with 0 μM bilirubin; B50, SHED treated with 50 μM bilirubin; B50+PAM, SHED treated with 50 μM bilirubin and 10 μM pamidronate. **d-f**: n = 5 for all groups. Statistical analysis was performed as described in the Methods. Graph bars showed the means ± SEM. $^{*}P < 0.05$, $^{**}P < 0.01$, and $^{***}P < 0.005$. NS, no significance. NS, no significance.
